# C1QTNF6 Overexpression Acts as a Predictor of Poor Prognosis in Bladder Cancer Patients

**DOI:** 10.1155/2020/7139721

**Published:** 2020-10-16

**Authors:** Xin Zhu, Hang Tong, Shun Gao, Hubin Yin, Gongmin Zhu, Xinyuan Li, Weiyang He, Xin Gou

**Affiliations:** Department of Urology, The First Affiliated Hospital of Chongqing Medical University, Chongqing 400016, China

## Abstract

**Background:**

Bladder cancer is one of the most common urinary malignancies. This study is aimed at providing some promising molecular biomarkers for bladder cancer (BC) by investigating the correlation between C1QTNF6 expression and clinical characteristics as well as prognosis in patients with bladder cancer.

**Methods:**

Sequencing profiles of C1QTNF6 mRNA in BC patients were collected to evaluate the distinctive gene expression, between normal bladder mucosa and BC, according to the TCGA and GEO databases. The association between C1QTNF6 expression and the clinical features as well as the disease prognosis was evaluated using two independent cohorts. The expression of C1QTNF6 in normal bladder and BC cells was examined by western blotting and PCR, so the underlying molecular mechanism could be further investigated.

**Results:**

C1QTNF6 mRNA levels were found to be differentially expressed in two independent public cohorts, including the TCGA database and GSE13507 dataset from GEO. The protein and RNA levels of C1QTNF6 in BC cells were both elevated when compared to normal bladder cell lines. High C1QTNF6 expression was detected in advanced T/M stages, pathological grade, and AJCC stage when compared to the low C1QTNF6 expression group. The underlying mechanism related to this differential expression could be explained by cell migration and invasion assays, where bladder cancer cells 5637 and T24 had a significant reduction on migration and invasion ability upon knockdown of C1QTNF6 expression. The low C1QTNF6 expression group presented a more prominent OS advantage over the high-expression group in both TCGA and GSE13507 cohorts. Moreover, the protein content in tissues was further validated using the HPA database and TMA. Survival analyses also indicated that the high C1QTNF6 expression group had an unfavorable OS when compared to the low-expression group.

**Conclusions:**

High C1QTNF6 expression may serve as a predictor of poor prognosis in bladder cancer patients, and the underlying mechanism is possibly associated with changes on cancer cell migration and invasion ability.

## 1. Introduction

Bladder cancer (BC) has been considered as the fourth most common type of cancer affecting men worldwide. In 2019, a total of 61,700 new cases and 12,870 deaths of male patients have been estimated in the United States [1]. This disease typically leads to less morbidity and mortality in women, with an estimate of 18,770 new cases and 4,800 deaths [1]. Clinical and histopathological characteristics, such as tumor stage, lymph node metastasis, and the presence of molecular markers such as epidermal growth factor receptor 3, play significant roles in predicting the prognosis of bladder cancer. Therefore, a more in-depth exploration of the molecular mechanisms related to bladder carcinogenesis, as well as the elucidation of novel BC biomarkers, may provide further strategies for the diagnosis, therapy, and prognosis of this malignancy [2].

C1QTNF6, as a member of the superfamily of C1q and tumor necrosis factor (C1QTNF), has been originally identified in digestive cancers. Among 30 liver cancer samples, 21 has been positive for C1QTNF6, while normal liver tissues are typically negative. Moreover, the recombinant protein C1QTNF6 appears to effectively increase the expression of Akt and promote liver cancer angiogenesis [3]. Additional studies have indicated that C1QTNF6 depletion may negatively impact the invasion and migration of liver cancer cells by inactivating the AKT pathway [4]. It has also been reported that C1QTNF6 is overexpressed in gastric cancer. Interestingly, C1QTNF6 knockout can significantly decrease the proliferation and metastatic ability of gastric cancer cells [5]. An increased C1QTNF6 secretion may eventually contribute to mitochondrial DNA loss, which is closely involved with tumorigenesis [6].

Considering the scarcity of studies focusing on the roles of C1QTNF6 in BC, we presently investigated the differential expression of C1QTNF6 in bladder cancer and normal tissues and further explored the underlying correlation between C1QTNF6 levels and clinical BC features (as well as prognosis), which altogether may shed light in understanding the biological functions of C1QTNF6 in this pathological condition.

## 2. Materials and Methods

### 2.1. Data Collection

RNA sequencing profiles and clinical data of bladder cancer were retrieved from The Cancer Genome Atlas (TCGA) data portal (https://portal.gdc.cancer.gov/) and the cBio Cancer Genomics Portal (http://cbioportal.org) in August 2019. Another independent dataset GSE13507 from the GEO database was also utilized to validate the differential expression of C1QTNF6 in cancerous and normal bladder tissues, as well as their characteristics and potential prognosis.

### 2.2. Cell Culture

A set of human BC cell lineages (T24, 5637, UMUC3, RT4, and BIU87) and a normal urothelial bladder cell line (SV-HUCL) were obtained from the American Type Culture Collection (ATCC, Manassas, VA, USA). T24, BIU87, and 5637 cells were cultured in RPMI 1640 medium (Corning Incorporated, Corning, NY, USA) supplemented with 10% fetal bovine serum (FBS, Gibco, Thermo Scientific, Waltham, MA, USA). UM-UC-3 cells were grown in Dulbecco's modified Eagle's medium (DMEM, Corning Incorporated, Corning, NY, USA) containing 10% FBS. RT4 cells were cultured in McCoy's 5A modified medium (Boster Biological Technology, Wuhan, China) containing 10% FBS. SV-HUCL cells were grown in F-12K medium (Thermo Scientific, Waltham, MA, USA) containing 10% FBS. All cells were maintained in a medium supplemented with 1% penicillin/streptomycin and incubated at 37°C in an atmosphere of 5% CO_2_.

Small interfering RNAs (siRNAs) targeting C1QTNF6 (siC1QTNF6; siRNA1, GCAACGACUUCGACACCUATT UAGGUGUCGAAGUCGUUGCTT; siRNA2, CCUGAUGUGUGAGAUCCCUTT AGGGAUCUCACACAUCAGGTT) were synthesized by GenePharma (Shanghai, China). Lipofectamine 3000 (Invitrogen, Thermo Scientific, Waltham, MA, USA) was used for cell transfection, according to the manufacturer's instructions.

### 2.3. Western Blotting

Cells were lysed in RIPA lysis buffer (Beyotime Institute of Biotechnology, Jiangsu, China) supplemented with 1% protease inhibitor (Sigma-Aldrich, Saint Louis, MO, USA). Protein concentration was determined using a BCA Protein Assay Kit (Beyotime Institute of Biotechnology, Jiangsu, China). Total protein (30-40 *μ*g per sample) was resolved by SDS-PAGE using a 10% gel electrophoresis. Thereafter, protein content was transferred to polyvinylidene difluoride (PVDF) membrane (Merck Millipore, Darmstadt, Germany) and then incubated with 5% nonfat milk in Tris-buffer saline containing 0.1% Tween 20 (TBST) for 1-2 hrs at room temperature. Next, the membrane was incubated overnight at 4°C with primary antibody against C1QTNF6 (ab36900, 1 : 1000 dilution, Abcam, USA) or GAPDH (AB0037, 1 : 5000 dilution, Abways, China). After washing with TBST, the membrane was then incubated with horseradish peroxidase- (HRP-) conjugated secondary antibodies (#7074, Cell Signaling Technology, USA). Respective protein signals were visualized by enhanced chemiluminescence (ECL) chromogenic substrate (Bio-Rad Laboratories, USA). GAPDH was used as a loading control.

### 2.4. Quantitative Reverse Transcription (qRT) PCR

Total RNA was extracted using Trizol reagent (Takara Biotechnology Co., Ltd., China). First-strand cDNA synthesis was performed using the PrimeScript 1st Stand cDNA Synthesis Kit (Takara Biotechnology Co., Ltd., China). Quantitative PCR (qPCR) was then conducted using quantitative PCR reagents SYBR Premix Ex TaqTM (Takara Biotechnology Co., Ltd., China). *β*-Actin expression was measured as an internal control. All mRNA levels were evaluated by the 2^-*ΔΔ*Ct^ method. All reactions were performed in triplicates.

### 2.5. In Vitro Migration and Invasion Assays

For the migration and invasion analysis, cells were seeded into the upper chambers of Transwells (Corning Incorporated, Corning, NY, USA) in a serum-free medium. For the migration assay, cells were harvested 48 hrs after transfection, resuspended in serum-free RPMI 1640 and then loaded into respective upper chambers (5 × 10^4^ cells/200 *μ*L). Lower chambers were loaded with 600 *μ*L of medium containing 10% FBS. The incubation time was set to 12 hrs for T24 cells and 24 hrs for 5637 cells. After washing with PBS, cells from the upper surface of the chamber were removed with a cotton swab. Cells from the lower surface of the membrane were fixed with 4% paraformaldehyde (PFA) for 15 mins and stained with a 0.1% crystal violet solution for another 15 mins. Five Transwell fields were randomly photographed with an inverted optical microscope. For the invasion assay, procedures followed according to the migration assay with the exception that a Transwell chamber with Matrigel was alternatively used.

### 2.6. Immunohistochemistry

Rabbit polyclonal antibody against C1QTNF6 was obtained from Sigma (College Park, MD, USA). A tissue microarray (TMA), consisting of 54 bladder cancer cases, was obtained from OUTDO BIOTECH (Shanghai, China). After deparaffination, rehydration, and antigen retrieval, the TMA slide was then incubated with primary rabbit polyclonal anti-human CTRP6 (dilution 1 : 200; Sigma Antibody; SAB487P) overnight at 4°C. Afterwards, the TMA slide was incubated with an anti-rabbit secondary antibody (ready-to-use solution; Cell Signaling Technology; #8114), followed by chromogen diaminobenzidine staining. The TMA slide was quantitatively scored according to the staining intensity and proportion using a microscope. The staining intensity of each specimen was scored as negative = 0, weakly positive = 1, moderate positive = 2, and strong positive = 3. The staining percentage was scaled as 1 (0–10%), 2 (11%–50%), 3 (50%–75%), or 4 (75%–100%). According to the immunohistochemical scores related to C1QTNF6 protein levels, specimens were divided into (i) a high-expression group (with a score greater than or equal to 4) and (ii) a low-expression group (with a score lower than 4).

### 2.7. Human Protein Atlas (HPA)

The Human Pathology Atlas (HPA) project (https://www.proteinatlas.org) includes immunohistochemistry data derived from a TMA-based analysis of 44 distinct tissue types, as well as the proteome analysis of 17 major malignancies [7, 8]. Immunostaining intensity and patient information (with the corresponding cancer types) were available online. In this report, a representative image of protein expression, using immunohistochemistry for C1QTNF6, was captured and then compared to both BC and normal bladder tissues from HPA.

### 2.8. Gene Set Enrichment Analysis

Gene set enrichment analysis (GSEA) was applied to evaluate the correlations between C1QTNF6 expression and relevant pathways, using the bladder cancer dataset from TCGA. The detailed protocol for GSEA is available on the Broad Institute gene set enrichment analysis website (http://www.broad.mit.edu/gsea). Datasets and phenotype label files were created and loaded onto the GSEA software (v 4.0.1; Broad Institute, Cambridge, USA). The samples were separated into a high group and a low group based on the median C1QTNF6 level. The analysis was randomly repeated 1,000 times. A meaningful gene set was defined as an adjusted *P* value < 0.05 and a false discovery rate (FDR) < 0.25. Statistical analysis and graphical plotting were conducted using the R software (version 3.3.2).

### 2.9. Statistical Analysis

The statistical difference between respective groups was evaluated by Student's *t*-test or one-way analysis of variance (ANOVA), using GraphPad Prism (GraphPad Software, CA, USA). Survival curves were determined by the Kaplan-Meier method. A *P* value < 0.05 was set to define statistical significance.

## 3. Results

### 3.1. C1QTNF6 mRNA Levels in BC and Normal Tissues

**Figure 1:**
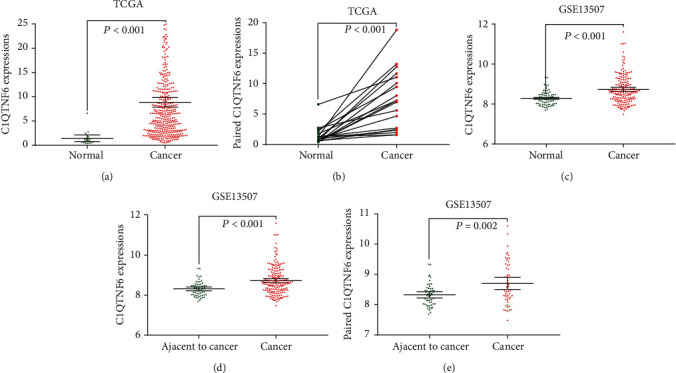
C1QTNF6 expression in normal and bladder cancer tissues. (a) Scatter plot from TCGA cohort indicating a higher expression of C1QTNF6 in bladder cancer when compared to normal tissues (*P* < 0.001). (b) Paired C1QTNF6 expression between normal and bladder cancer tissues from TCGA cohort (*P* < 0.001). (c) Scatter plot from GSE13507 cohort indicating a higher expression of C1QTNF6 in bladder cancer when compared to normal tissues (*P* < 0.001). (d) Scatter plot from GSE13507 cohort indicating higher expression of C1QTNF6 in bladder cancer when compared to adjacent normal tissues (*P* < 0.001). (e) Paired C1QTNF6 expression between adjacent normal and bladder cancer tissues from GSE13507 cohort (*P* = 0.002).

To compare the expression levels of C1QTNF6 in cancerous and normal bladder tissues, we first analyzed RNA-Seq data related to C1QTNF6 in the TCGA-based BLCA cohort. Both normal (*n* = 19) and cancerous (*n* = 411) bladder tissues were included to properly compare C1QTNF6 mRNA levels. As shown in Figure 1(a), C1QTNF6 expression was significantly elevated in BC when compared to normal bladder tissues. A paired comparison between the 19 BC samples and the corresponding normal tissues also demonstrated a significantly higher expression of C1QTNF6 in BC (Figure 1(b)). To further evaluate the differential expression of C1QTNF6 in other groups, another independent cohort was evaluated. For this, 67 normal bladder tissues (including 9 normal bladder mucosa from patients with benign disease and 58 adjacent normal bladder tissues from BC patients) and cancerous (*n* = 188) bladder tissues were compared, and an elevated C1QTNF6 expression was noticed again in BC (Figure 1(c)). Next, adjacent normal bladder tissues (*n* = 58), derived from BC patients, were compared with BC tissues (*n* = 188). Again, a higher expression of C1QTNF6 was detected in BC (Figure 1(d), *P* < 0.001). Moreover, a comparative analysis of C1QTNF6 levels in BC tissues (*n* = 52) and paired adjacent normal tissues was further conducted, reiterating the elevated C1QTNF6 expression in BC (Figure 1(e), *P* < 0.002).

### 3.2. C1QTNF6 Expression In Vitro

**Figure 2:**
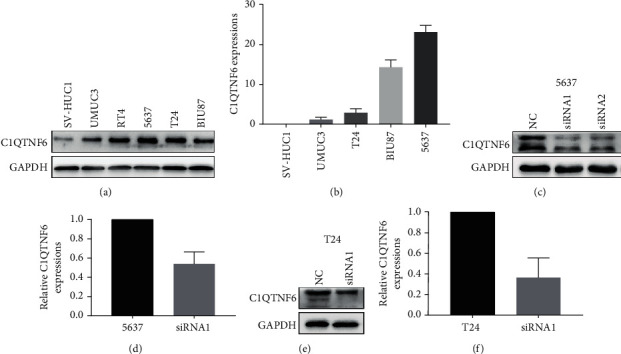
C1QTNF6 protein and RNA levels in vitro. (a) C1QTNF6 protein levels in normal bladder cells (SV-HUC1) and BC cell lines (UMUC3, RT4, 5637, T24, and BIU87). (b) C1QTNF6 RNA levels in normal bladder cells (SV-HUC1) and BC cell lines (UMUC3, T24, BIU87, and 5637). (c) Knockdown effects, mediated by siRNAs against C1QTNF6, towards protein levels in BC 5637 cells. (d) Knockdown effects, mediated by siRNAs against C1QTNF6, towards mRNA levels in BC 5637 cells. (e) Knockdown effects, mediated by siRNAs against C1QTNF6, towards protein levels in BC T24 cells. (f) Knockdown effects, mediated by siRNAs against C1QTNF6, towards mRNA levels in BC T24 cells.

Although the expression of C1QTNF6 was explored in tissues derived from two different cohorts, the expression analysis of C1QTNF6, at the cellular level, was still warranted. For this, the levels of C1QTNF6 protein were evaluated in a subset of BC cell lines, including UMUC3, RT4, 5637, T24, and BIU87, and a normal bladder cell lineage (SV-HUCL) (Figure 2(a)). Analysis of RNA expression was also conducted, indicating that the relative expression of C1QTNF6 in UMUC3, T24, BIU87, and 5637 was significantly elevated when compared to the levels detected in normal bladder cells (Figure 2(b)).

### 3.3. Correlation between C1QTNF6 Expression and Clinical Characteristics in BC Patients

**Figure 3:**
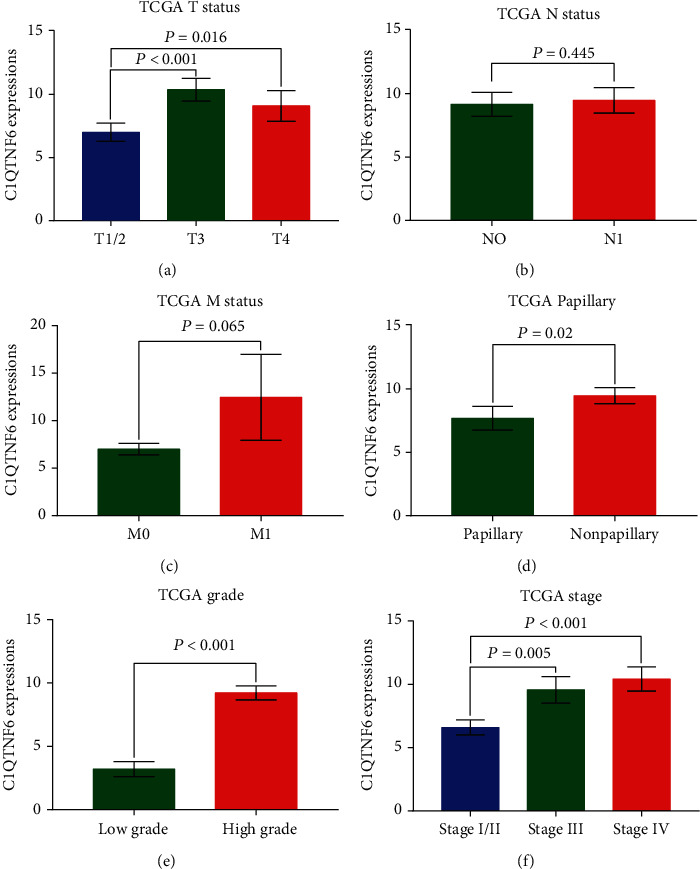
Association between C1QTNF6 expression and clinical characteristics of the TCGA cohort. (a) Distribution of C1QTNF6 expression stratified by T status. Bar graph indicates that a higher C1QTNF6 expression was associated with T3 (vs. T1/2, *P* < 0.001) and T4 (vs. T1/2, *P* = 0.016). (b) Bar graph indicating no significant association between C1QTNF6 expression and N1 (vs. N0, *P* = 0.445). (c) Bar graph indicating a trend for higher C1QTNF6 expression in M1 with no statistical significance (vs. M0, *P* = 0.065). (d) Bar graph indicating higher C1QTNF6 expression associated with the nonpapillary morphological type (vs. papillary type, *P* = 0.02). (e) Bar graph indicating higher C1QTNF6 expression in high tumor grade, when compared with low grade (*P* < 0.001). (f) Bar graph indicating higher C1QTNF6 expression in stages III (vs. stage I/II, *P* = 0.005) and IV (vs. stage I/II, *P* < 0.001).

**Figure 4:**
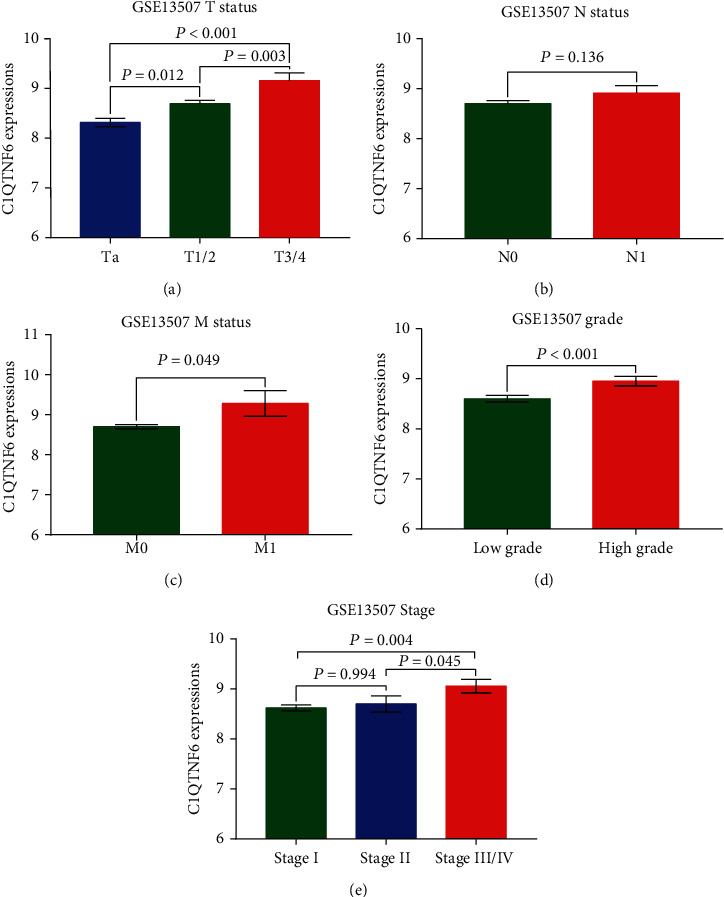
Association between C1QTNF6 expression and clinical characteristics of the GSE13507 cohort. (a) Distribution of C1QTNF6 expression stratified by T status. Bar graph indicates that a higher C1QTNF6 expression was associated with T1/2 (vs. Ta, *P* = 0.012) and T3/4 (vs. Ta, *P* < 0.001; vs. T1/2, *P* = 0.003). (b) Bar graph indicating no significant association between C1QTNF6 expression and N1 (vs. N0, *P* = 0.136). (c) Bar graph indicating higher C1QTNF6 expression in M1 (vs. M0, *P* = 0.049). (d) Bar graph indicating that elevated C1QTNF6 expression is associated with high tumor grade, when compared with low grade (*P* < 0.001). (e) Bar graph indicating higher C1QTNF6 expression in stage III/IV (vs. stage I, *P* = 0.004; vs. stage II, *P* = 0.045).

To explore the underlying correlation between C1QTNF6 RNA levels and clinical features of BC patients, the clinical information of 413 affected individuals was further accessed. For this, some clinical variables including TMN status, presence/type of papillary growth, and histological grade/stage were used to evaluate the potential association between C1QTNF6 expression and the clinical characteristics of BC patients. More elevated C1QTNF6 expression was observed in cases with advanced T status (Figure 3(a)), nonpapillary growth type (Figure 3(d)), pathological grade (Figure 3(e)), and AJCC stage (Figure 3(f)), with no association with positive lymph nodes (Figure 3(b)), and M status (Figure 3(c)). A validation analysis in GSE reiterated a strict association between high C1QTNF6 expression and advanced T status (Figure 4(a)), M status (Figure 4(c)), pathological grade (Figure 4(d)), and AJCC stage (Figure 4(e)). Again, no positive association with lymph node positiveness and C1QTNF6 expression was detected (Figure 4(b)).

### 3.4. Correlation between C1QTNF6 RNA Levels and Cell Migration/Invasion

**Figure 5:**
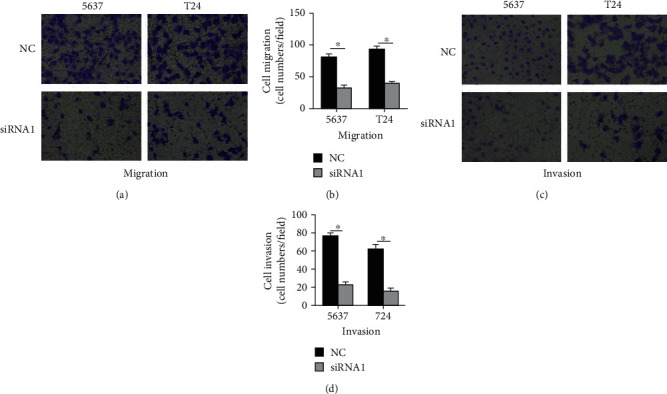
Depletion of C1QTNF6 expression decreases the invasion and migration of 5637 and T24 cells. (a) Migration assays of 5637 and T24 cells expressing control and C1QTNF6-specific siRNAs (NC and siRNA1, respectively). ^∗^*P* < 0.05; ^∗∗^*P* < 0.01. (b) Activity of migrating cells from respective groups of 5637 and T24 cells. (c) Invasion assays of 5637 and T24 cells expressing control and C1QTNF6-specific siRNAs (NC and siRNA1, respectively). ^∗^*P* < 0.05; ^∗∗^*P* < 0.01. (d) Activity of invading cells from respective groups of 5637 and T24 cells.

Considering the close association between RNA levels and TM status, the underlying correlation involving C1QTNF6 expression was evaluated in 5627 and T24 cell lines. For this, Transwell cell migration and invasion assays were further performed. According to the migration assays, the number of migrating 5637siRNA1 and T24siRNA1 cells was significantly decreased when compared with the control groups (i.e., 5637 and T24 NC cells) (Figures 5(a) and 5(b)). Consistently, invasion assays also indicated a significant reduction in the content of invading cells from the 5637siRNA1 and T24siRNA1 groups when compared with respective control groups (Figures 5(c) and 5(d)).

### 3.5. Gene Set Enrichment Analysis (GSEA)

**Figure 6:**
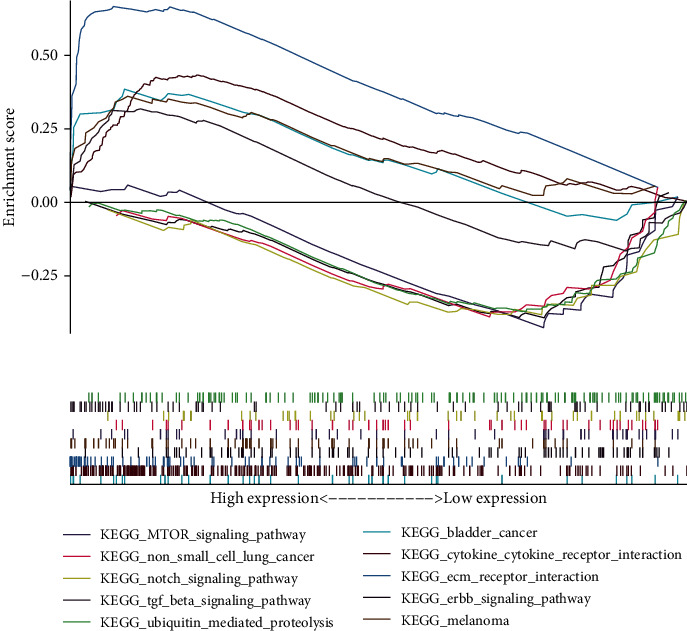
GSEA of C1QTNF6 expression in the TCGA cohort. The high-expression group was enriched in networks related to bladder cancer, cytokine-cytokine receptor interaction, ECM receptor interaction, ERBB signaling pathway, and melanoma. The low-expression group was enriched in pathways related to the mTOR signaling pathway, non-small-cell lung cancer, Notch signaling pathway, TGF-*β* signaling pathway, and ubiquitin-mediated proteolysis.

RNA-Seq data originated from BC patients with different levels of C1QTNF6 expression were presently compared. Respective results indicated that, in patients with high C1QTNF6 levels, genes that were enriched in particular networks, including bladder cancer, cytokine-cytokine receptor interaction, ECM receptor interaction, ERBB signaling pathway, and melanoma, varied significantly (Figure 6). In contrast, genes related to low C1QTNF6 expression were enriched in the mTOR signaling pathway, non-small-cell lung cancer, Notch signaling pathway, TGF-*β* signaling pathway, and ubiquitin-mediated proteolysis (Figure 6).

### 3.6. High C1QTNF6 Expression Predicts Poor OS and PFS in BC Patients

**Figure 7:**
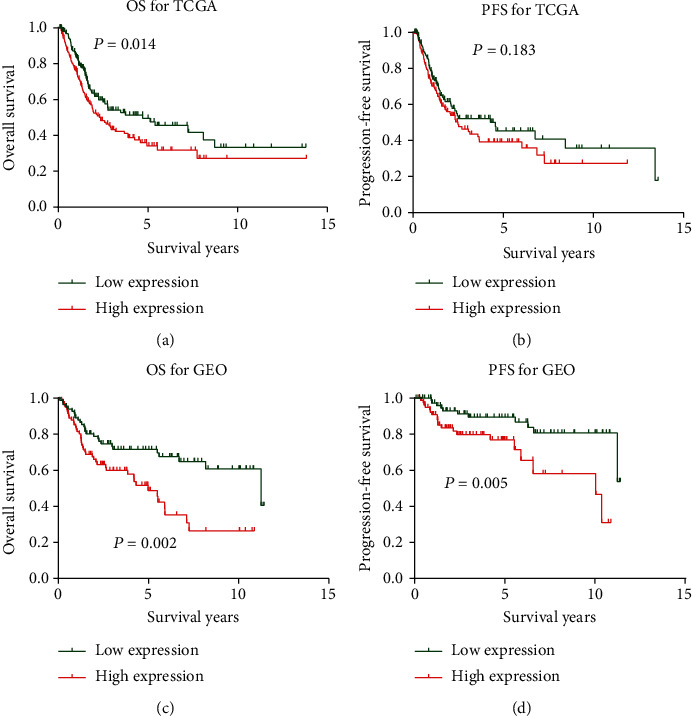
Kaplan-Meier survival curves for C1QTNF6 in TCGA and GSE13507 cohorts. (a) Kaplan-Meier survival curve of overall survival for C1QTNF6 in TCGA cohort (*P* = 0.014); (b) Kaplan-Meier survival curve of progression-free survival for C1QTNF6 in TCGA cohort (*P* = 0.183); (c) Kaplan-Meier survival curve of overall survival for C1QTNF6 in GSE13507 cohort (*P* = 0.002); (d) Kaplan-Meier survival curve of progression-free survival for C1QTNF6 in GSE13507 cohort (*P* = 0.005).

A total of 395 BC patients with detailed follow-up information were included for survival analysis. Patients were divided into low-expression (*n* = 198) and high-expression (*n* = 90) groups, according to their median levels of C1QTNF6 expression. As shown in Figure 7(a), the overall survival (OS) of the low-expression group was statistically more favorable than the high-expression group. Due to eventual dropouts and/or missing data for the evaluation of progression-free survival (PFS), only 310 patients were included for further comparison between the low- and high-expression groups. Although the low C1QTNF6 expression group presented some advantageous PFS over the high-expression group, this difference had no statistical significance (Figure 7(b), *P* < 0.183). Using a validation cohort from GSE, the median expression of C1QTNF6 was set as a cut-off for both OS and PFS. In this case, the low-expression group presented some significant OS and PFS advantages when compared to the high-expression group (Figure 7(c) for OS, *P* = 0.002; Figure 7(d) for PFS, *P* = 0.005).

### 3.7. Correlation between C1QTNF6 Protein Levels and Clinical Features of BC Patients

**Figure 8:**
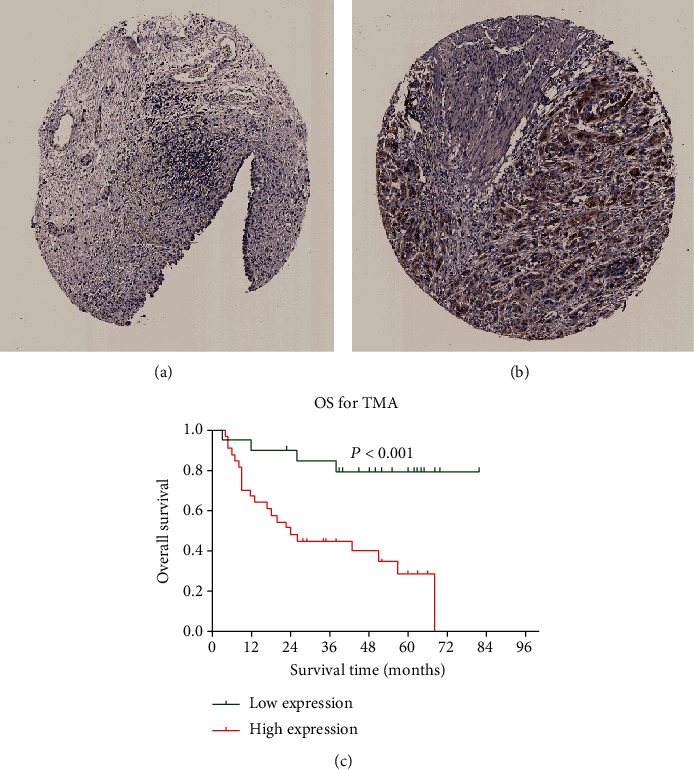
(a) Protein levels of C1QTNF6 in normal bladder mucosa from the HPA database. (b) Protein levels of C1QTNF6 in bladder cancer from the HPA database. (c) Survival analysis indicating that the high C1QTNF6 expression group is associated with a less favorable OS when compared to the low-expression group.

**Figure 9:**
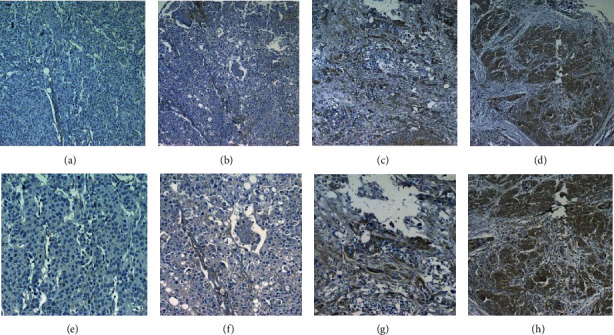
Representative immunohistochemical staining of C1QTNF6 expression. (a, e) Negative staining (score 0) in BC tissues. (b, f) Weakly positive staining in BC tissues. (c, g) Positive staining in BC tissues. (d, h) Strongly positive staining (score 2) in BC tissues. Magnification, ×100 and ×200.

Protein data related to C1QTNF6 levels was retrieved from the HPA database. As illustrated in Figures 8(a) and 8(b), the normal bladder mucosa was negative for C1QTNF6 while BC tissues were positive. Thereafter, we conducted a TMA-based IHC where we retrieved 54 bladder cancer cases with detailed follow-up information. Based on the IHC scores, the whole data was divided into low and high C1QTNF6 expression groups. The clinical characteristics of the respective groups are summarized in Supplementary Table 1. The typical IHC results were divided after multiplying respective staining and identified areas (Figure 9). Survival analysis also indicated that high C1QTNF6 expression could lead to an unfavorable OS in BC patients, when compared to low C1QTNF6 expression (Figure 8(c)).

## 4. Discussion

Approximately 70% of the newly diagnosed BC cases are classified as non-muscle-invasive bladder cancers. Therapeutically, transurethral resection of bladder tumors has been frequently combined with intravesical chemotherapy or Bacillus Calmette-Guerin immunotherapy. In regard to muscle-invasive BC, radical cystectomy still remains the most effective treatment; however, short- and long-term complications following this procedure have been closely related to the patient prognosis [9]. The modified Charlson comorbidity index has been widely used to predict perioperative mortality and long-term overall survival of patients with muscle-invasive BC undergoing radical cystectomy [10]. Despite the continuous improvements in the surgical methods towards BC, in addition to chemotherapy, immunotherapy, and other therapeutic approaches, the treatment efficacy has been solely improved to a certain extent. As a result, postoperative recurrence and metastasis are still quite common, with no significant improvement on 5-year survival rates [11].

The protein family composed by C1QTNF, adiponectin, leptin, and cerebellar peptide has been also named as the adipose factor superfamily [12]. The C1QTNF family of proteins is characterized by a highly conserved C-terminal complementary C1q domain. C1QTNF-related proteins (CTRPS) mainly consisted of 16 members, including C1QTNF 1-9, 9b, 10-15 [13]. All members are secreted proteins, widely expressed in a variety of tissues and cell types, which are involved in a variety of biological processes, such as host defense, inflammation, apoptosis, autoimmunity, cell differentiation, organogenesis, hibernation, and insulin-resistant obesity [14–19]. C1QTNF6, as a member of the C1QTNF family, has been shown to be associated with carcinogenesis in digestive cancers. However, the biological functions of C1QTNF6 in bladder cancer (BC) have been very limited.

Our current study initially explored the differential expression of C1QTNF6 in two independent public cohorts, based on the TCGA database and GSE13507 dataset from GEO. Compared with normal bladder mucosa and paired adjacent tissues, BC was remarkably associated with C1QTNF6 overexpression. Regarding the levels of C1QTNF6 protein and RNA in vitro, both were significantly elevated in BC cells when compared to a normal bladder cell line. Further analyses focused on the correlation between C1QTNF6 expression and clinical characteristics of BC patients indicated that high C1QTNF6 expression was more prominent in advanced TM status as well as pathological and AJCC stages when compared with the low-expression group. The underlying mechanism related to this differential expression could be better explained by further migration and invasion assays, where BC cells 5637 and T24 presented a significant reduction on migration and invasion capabilities upon knockdown of C1QTNF6 expression. The putative cancer-related pathways related to the differential C1QTNF6 expression included (i) cytokine-cytokine receptor interaction, (ii) ECM receptor interaction, (iii) ERBB signaling pathway, (iv) mTOR signaling pathway, (v) Notch signaling pathway, (vi) TGF-*β* signaling pathway, and (vii) ubiquitin-mediated proteolysis. Moreover, this study investigated the predictive value of C1QTNF6 expression in the prognosis of BC patients. We were able to demonstrate that a low C1QTNF6 expression group had a more advantageous OS when compared to the high-expression group according to both TCGA and GSE13507 cohorts. Still, no statistical significance in PFS was only found in the GSE13507 cohort. Afterwards, the content of C1QTNF6 protein in tissues was validated by TMA and HPA database analysis. According to the IHC scores of TMA, a higher expression of C1QTNF6 appears to provide a more unfavorable OS when compared to the low-expression group.

Nevertheless, some limitations were still noticed in this study. Firstly, the difference in gene expression and prognosis evaluation was analyzed using some retrospective data. In addition, the molecular mechanisms involved in the elevated expression of C1QTNF6 in BC may require further investigation of related cancer pathway(s) as well as in vivo analyses. Finally, the limited sample number of BC patients and different datasets may lead to some heterogeneity of the data analysis.

## 5. Conclusions

Here, we demonstrated that C1QTNF6 is significantly increased, at both RNA and protein levels, in BC cells and tissues. Moreover, higher C1QTNF6 expression can predict a worse prognosis for BC patients. The increased cell invasion and migration ability associated with high C1QTNF6 expression may better explain the biological impact of C1QTNF6 in BC progression.

## Data Availability

The authors declare that the data supporting the findings of this study is available within the article.

## Abbreviations

C1QTNF: C1q and tumor necrosis factor
TCGA: The Cancer Genome Atlas
FBS: Fetal bovine serum
DMEM: Dulbecco's modified Eagle's medium
siRNAs: Small interfering RNAs
TBST: Tris-buffer saline with 0.1% Tween
HRP: Horseradish peroxidase
ECL: Enhanced chemiluminescence
TMA: Tissue microarray
HPA: Human Pathology Atlas
GSEA: Gene set enrichment analysis
FDR: False discovery rate
ANOVA: Analysis of variance
OS: Overall survival
PFS: Progression-free survival.

## Supplementary Materials

Supplementary Materials Supplementary Table 1. The clinical characteristics of TMA Cohort.
