# Supplementary material for: C1QTNF6 Overexpression Acts as a Predictor of Poor Prognosis in Bladder Cancer Patients
Source: Biomed Res Int. 2020 Oct 16;2020:7139721. doi: 10.1155/2020/7139721 (PMC7585664; doi:10.1155/2020/7139721)
Supplement: Supplementary Materials — Supplementary Table 1. The clinical characteristics of TMA Cohort. [file 7139721.f1.docx]

| **Supplementary Table 1. The clinical characteristics of TMA Cohort** | | | |  |
| --- | --- | --- | --- | --- |
| **Variables** | **Number** | **Low CTRP6（n=21）** | **High CTRP6（n=33）** |  |
|  |  |  |  |  |
| **M Status** |  |  |  |  |
| M0 | 54 | 20 | 34 |  |
| M1 | 0 | 0 | 0 |  |
| **Age** |  |  |  |  |
| ≤ 60 | 12 | 2 | 10 |  |
| 60-70 | 14 | 8 | 6 |  |
| >70 | 28 | 11 | 17 |  |
| **N Status** |  |  |  |  |
| No | 37 | 15 | 22 |  |
| Yes | 6 | 1 | 5 |  |
| Unknown | 11 | 5 | 6 |  |
| **T Status** |  |  |  |  |
| Tis | 5 | 3 | 2 |  |
| T1 | 10 | 6 | 4 |  |
| T2 | 14 | 7 | 7 |  |
| T3 | 21 | 4 | 17 |  |
| T4 | 2 | 0 | 2 |  |
| Unknown | 2 | 1 | 1 |  |
| **Grade** |  |  |  |  |
| Low | 2 | 2 | 0 |  |
| High | 52 | 19 | 33 |  |
| **Sex** |  |  |  |  |
| Female | 8 | 2 | 6 |  |
| Male | 46 | 19 | 27 |  |
